# Association between Charlson comorbidity index score and outcome in patients with stage IIIB-IV non-small cell lung cancer

**DOI:** 10.1186/s12890-017-0452-0

**Published:** 2017-08-15

**Authors:** Lei Zhao, Lai-Han Leung, Jing Wang, Huihui Li, Juanjuan Che, Lian Liu, Xiaojun Yao, Bangwei Cao

**Affiliations:** 1 Cancer Center, Beijing Friendship Hospital, Capital Medical University, 95 Yong An Road, Xicheng District, Beijing, 100050 China; 2 State Key Laboratory of Quality Research in Chinese Medicine, Macau Institute for Applied Research in Medicine and Health, Macau University of Science and Technology, Taipa, Macau, 999078 China

**Keywords:** Charlson comorbidity, Index score, Outcome, Advanced NSCLC

## Abstract

**Background:**

This retrospective study investigated the association between the Charlson comorbidity index (CCI) score and the survival of patients with stage IIIB-IV (advanced, non-resectable) non-small cell lung cancer (NSCLC) who also did not have gene mutations in epidermal growth factor receptor (EGFR) or anaplastic lymphoma kinase (ALK).

**Methods:**

The records of 165 patients (28–80 y, median 61 y) who met the above criteria and were admitted to Beijing Friendship Hospital Capital Medical University from 1 May 2010 to 1 October 2014were reviewed. Associations between baseline variables and the CCI score were assessed via univariate and multivariate logistic regression analysis. Overall survival was defined as the time from the first clinic visit to death from any cause, or to the end of follow-up. Survival curves were estimated via the Kaplan-Meier method and compared with the log-rank test.

**Results:**

Logistic regression analyses indicated that smoking and performance status were independently associated with the CCI score. Smoking was associated with an increased risk of mortality (odds ratio (OR) 4.12 (95% confidence interval [CI] 1.92–8.84) compared to non-smokers), as was performance status 2 (ambulatory, capable of self-care, unable to perform any work activities; active for >50% of waking hours) (OR 2.22 (95% CI, 1.14–4.33) compared to performance status 1). Univariate Cox’s regression analyses showed that the hazard ratios were significantly associated with the CCI score (*P* = 0.009), smoking (*P* = 0.042), and male gender (*P* = 0.021).

**Conclusion:**

The CCI score is an important prognostic factor for the prediction of overall survival in patients with stage IIIB-IV NSCLC who are negative for EGFR and ALK gene mutations.

## Background

Non-small cell lung cancer (NSCLC) is a common malignant tumour worldwide [1]. Because there are few valid diagnoses of early-onset NSCLC, most patients have late stage cancer at the time of diagnosis [2, 3], and systemic chemotherapy or precision therapy with several targeted drugs are the only treatment options. According to a number of studies, the primary prognostic factors for NSCLC patients are pathological stage, performance status, gender, age, histology, and weight [4–7]. Because many patients are elderly at the time of diagnosis and because the prevalence of previous or current smokers is high, medical comorbidities are often present. Asmis et al. suggested that the presence of comorbidity, rather than being older than65 years, was associated with worse survival of NSCLC patients [8].

The use of comorbidities for prognostic assessment has been extensively studied in many fields [9, 10], including oncology [11–13]. A common model of comorbidity is the Charlson comorbidity index (CCI) [14]. The CCI is based on 19 underlying diseases with variously assigned weights that are combined into a composite score. The CCI was originally derived for hospitalized patients in general (internal) medicine, but revised versions have since been validated in multiple patient populations, including patients with NSCLC [15–18].

The CCI score has been positively associated with increased risk of death in patients with inoperable NSCLC treated with radiofrequency ablation (hazard ratio [HR] = 1.3, 95% confidence interval [CI; 25.5–58.2]) [16]. In patients with stages I-III NSCLC, the CCI score, gender, ethnicity, tumour stage, histology, and insurance type have been associated with overall survival; patients with higher CCI scores were found to have worse clinical outcomes [19]. The CCI score has also been found to be a better predictor of comorbidity than are individual risk factors in patients who have undergone resection for NSCLC [20].

The effects of treatment selection on outcome in both surgical and nonsurgical patients with early-stage NSCLC have been assessed [21]. However, to the best of our knowledge, there has been no study of a correlation between the CCI score and chemotherapy benefit or survival of patients with unresectable advanced NSCLC.

Elderly patients are often excluded from clinical trials because it is believed that they are unable to tolerate aggressive chemotherapy and are more likely to suffer increased toxicity, resulting in poorer quality of life. However, it has been demonstrated that age alone is not an independent prognostic factor, and the tolerance to treatment, its toxicity and the benefits experienced by elderly patients with good performance status are similar to those of the nonelderly [22].

The present study retrospectively investigated whether CCI scores can predict the long-term survival of patients with unresectable advanced NSCLC.

## Methods

The Research Ethics Board of Beijing Friendship Hospital approved this retrospective study. The study complied with the Declaration of Helsinki. All subjects gave written informed consent.

### Patient population

All subjects in this study conformed to the following criteria: visited an oncology clinic at Beijing Friendship Hospital, Capital Medical University (Beijing, China) between May 2010 and October 2014; aged ≥18 y; tested negative for both epidermal growth factor receptor (EGFR) and anaplastic lymphoma kinase (ALK) gene mutations; had unresectable NSCLC, stage IIIB-IV, which was confirmed histologically or cytologically and via imaging; and underwent a chemotherapy regimen, but not radiotherapy. The date of the last follow-up was 1 May 2015. We excluded patients who had previously received radiotherapy in the lung area, or who had an Eastern Cooperative Oncology Group (ECOG) performance status of≥ 3 [23].

Age, gender, histology, stage, ECOG performance status, and smoking status were obtained from the Beijing Friendship Hospital medical records (Table 1). The first clinic visit date was our index date, considered the time at which stage was established and from which survival time was measured. Cancer stage was defined in accordance with the 7^th^ edition of the Union for International Cancer Control (UICC) and the American Joint Committee on Cancer (AJCC) TNM staging system for stages I, II, III, and IV [24].

**Table 1:** Patient baseline characteristics^a^

			CCI < 9	CCI ≥ 9
Patients		165	90 (55%)	75 (45%)
Age, y		61 ± 11	60 ± 9	62 ± 10
Gender	Male	89 (54)	49 (55%)	40 (53%)
	Female	76 (46)	41 (45%)	35 (47%)
Histology	Non-squamous	92 (56)	50 (56%)	42 (56%)
	Squamous	73 (44)	40 (44%)	33 (44%)
Stage	IIIB	45 (27)	26 (29%)	19 (25%)
	IV	120 (73)	64 (71%)	56 (75%)
ECOG^b^	1	74 (45)	40 (44%)	34 (45%)
	2	91 (55)	50 (56%)	41 (55%)
Smoking status	Smoker	95 (58)	50 (56%)	45 (60%)
	Non-smoker	70 (42)	40 (44%)	30 (40%)

^a^ *n* (%) or mean ± standard deviation;

^b^There was no patient with performance status = 0 in our cohort

The CCI parameters and scores were assigned according to the definitions established by Charlson et al. [25]. Specifically, the CCI index assigns scores as follows. One point each is given for peripheral vascular disease, a history of myocardial infarction, heart failure, dementia, cerebrovascular disease, chronic pulmonary disease, connective tissue disorder, peptic ulcer disease, mild liver disease, and diabetes mellitus without complications. Two points are assigned for leukaemia, lymphoma, hemiplegia, moderate-to-severe renal disease, diabetes mellitus with complications, tumours without metastases, and myeloma. Three points are given for moderate or severe liver disease. Six points are assigned for acquired immunodeficiency syndrome or metastatic solid tumours.

We obtained additional comorbidity data via are view of the hospital administrative databases for individual patients, from both paper and electronic medical records. For conditions that could be scored in multiple categories based on disease severity, points were only counted for the more severe condition. For advanced NSCLC patients, cancer metastasis is common, and the CCI score was usually >6. The best cut-off value for the CCI and the survival data for all patients were evaluated through receiver operating characteristic (ROC) analysis.

### Statistical analysis

The outcome measure was overall survival, defined as the time from the first clinic visit date to death from any cause, or to the end of the follow-up period. Summaries of discrete variables are displayed in terms of proportion, and continuous variables are presented as means with 95% confidence intervals (95% CI). ROC analysis was used to determine the best cut-off value for the CCI and survival data for all the patients. Survival curves were estimated via the Kaplan-Meier method. Univariate and multivariate Cox’s regression analyses were used to determine the association between baseline characteristics (age, gender, histology, stage of cancer, CCI score, ECOG performance status, smoking status) and clinical outcomes. The survival curves were estimated via the Kaplan-Meier method. Logistic regression and multinomial logistic regression analyses were used to test the CCI score and baseline characteristics (gender, age, histology, tumour stage, ECOG performance status, smoking status). Significance was defined at *P* < 0.05. All statistical analyses were performed with SPSS 13.0 for Windows software (SPSS, Chicago, IL, USA).

## Results

From 1 May 2010 to 1 October 2014, 297 patients received a new diagnosis of stage IIIB-IV NSCLC. Of these, 132 patients were excluded for being positive for EGFR or ALK gene mutations, having received radiotherapy in the lung area, having a performance status level of ≥3, or being lost early during follow-up. Therefore, 165 patients were included in the analysis (Table 1).

The median follow-up duration for the surviving patients was 28 months; the last follow-up assessment was 1 May 2015. The median age of the patients was 61 years; 58% of the patients were smokers, and 56% and 44% had non-squamous or squamous carcinoma, respectively. The most prevalent comorbid illnesses (present in > 5% of the population each) were metastatic solid tumours (82%), chronic pulmonary disease (37%), mild liver disease (29%), cerebrovascular disease (23%), diabetes with end-organ damage (21%), congestive heart failure (21%), peptic ulcer disease (19%), and peripheral vascular disease (16%).

**Fig. 1:**
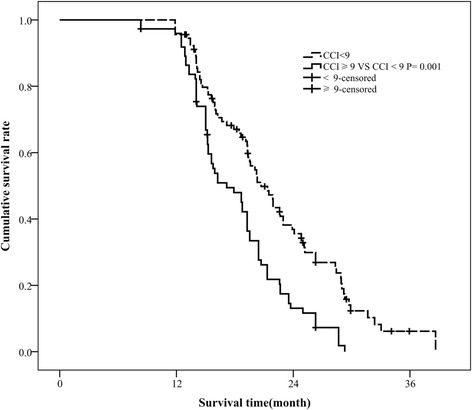
ROC curve for best cut-off of unresectable stage IIIB-IV NSCLC patients without EGFR or ALK genetic mutations. AUC, area under curve

Using ROC analysis, a CCI score of ≥9 was significantly associated with the decreased survival of patients in palliative care (*P* < 0.05). The result of ROC analysis showed that a CCI score of 9 was the cut-off (Fig. 1). Hence, for further analyses, we divided CCI scores as follows: CCI < 9 (moderate), indicating that patients have metastatic cancer or other complications; and CCI ≥ 9 (severe), indicating that patients have more complications.

**Fig. 2:**
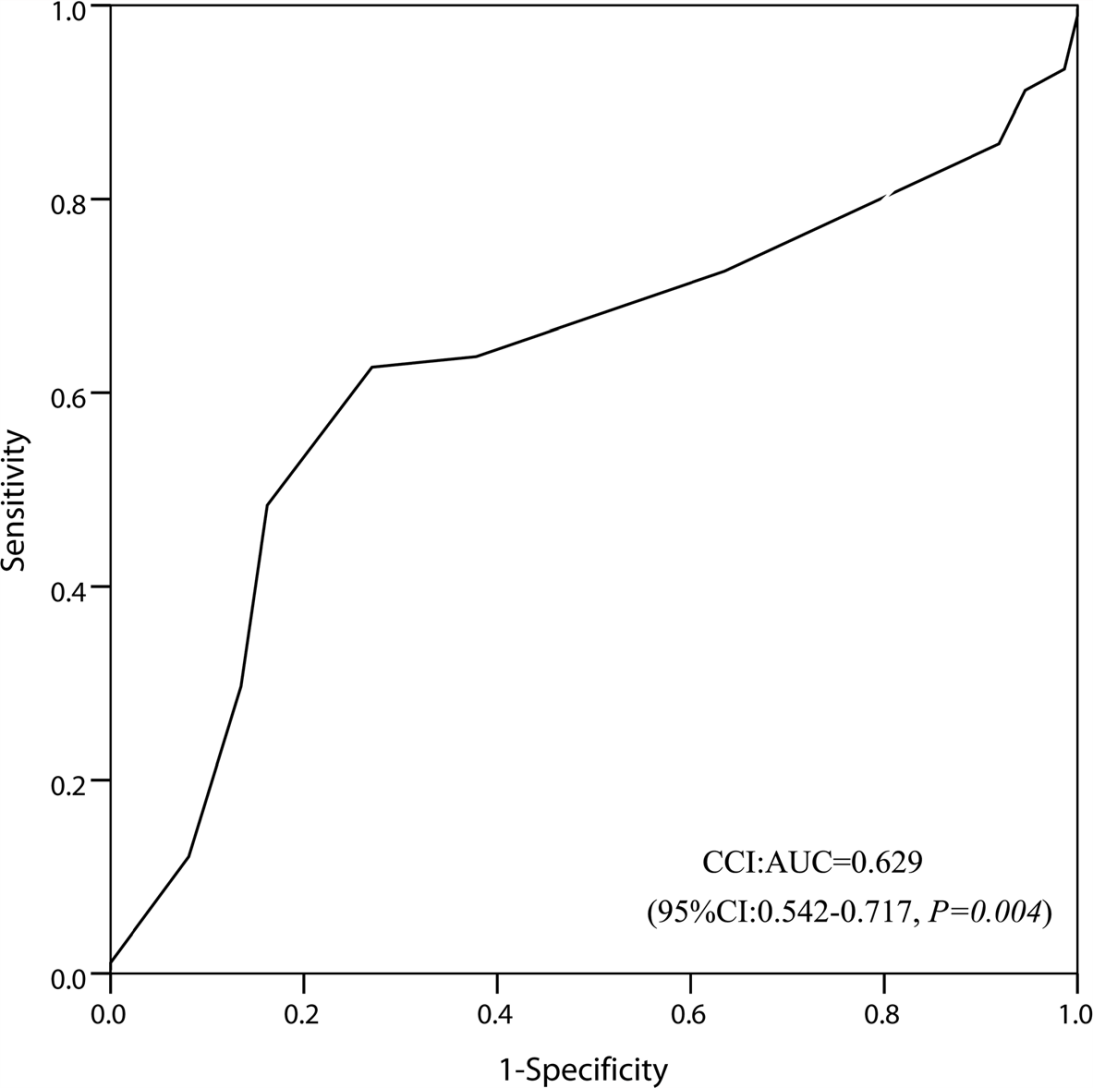
Kaplan-Meier curves for unresectable stage IIIB-IV NSCLC patients without EGFR or ALK genetic mutations, according to CCI score

Using modified CCI categories, CCI scores of <9 were classified as low, and CCI scores of ≥9 were considered high (90 and 75 subjects, respectively). The median survival time was 19.3 months (95% CI 17.00–23.30). The median survival rates at 1, 2, and 3 years were 78.31%, 41.56%, and 11.53%, respectively. The survival rates of patients in the low CCI score group at 1, 2, and 3 years (81.23%, 43.46%, and 16.45%, respectively) were better than those of patients with high CCI scores (62.48%, 38.79%, and 7.48%, respectively). The median survival time of patients in the low CCI score group (20.84 months [95% CI, 19.52–22.17]) exceeded that of patients in the high CCI score group (17.80 months [95% CI, 16.61–18.96]) (Fig. 2).

The logistic regression analyses showed that smoking status and performance status were independent prognostic factors for CCI scores (Table 2). Smoking and a higher performance status were associated with an increased risk of mortality, with smokers having an odds ratio (OR) of 4.12 (95% CI, 1.92–8.84), and patients with a performance status of 2 having an OR of 2.22 (95% CI, 1.14–4.33). Other factors (age, gender, histology, and tumour staging) were not significant (*P* > 0.05). The Cox regression analysis revealed that there were significant differences (*P* < 0.05) between stage, CCI score, smoking status, gender, and the probability of surviving.

**Table 2:** Association between CCI and baseline characteristic^a^

		Univariate analysis		Multivariate analysis	
		OR (95% CI)	*P*	OR (95% CI)	*P*
Age, y	< 65	1		1	
	≥65	1.73 (0.93–3.22)	0.082	1.30 (0.66–2.55	0.451
Gender	Male	1		1	
	Female	1.40 (0.75–2.59)	0.291	2.02 (0.96–4.27)	0.065
Histology	Adenocarcinoma	1		1	
	Squamous	1.03 (0.55–1.90)	0.935	0.89 (0.43–1.83)	0.748
Cancer stage	IIIB	1		1	
	IV	1.31 (0.65–2.63)	0.444	1.09 (0.50–2.38)	0.827
Smoking status	Nonsmoker	1		1	
	Smoker	3.53 (1.82–6.87)	<0.001	4.12 (1.92–8.84)	<0.001
Performance status^b^	1	1		1	
	2	2.37 (1.27–4.44)	0.007	2.22 (1.14–4.33)	0.019

^a^Odds ratio > 1 indicates greater likelihood of higher CCI; ^b^there was no patient with performance status = 0 in our cohort

Higher stages (stage IV), high CCI scores (CCI ≥ 9), smoking, and male gender were associated with higher HRs than were lower stages, lower CCI scores, not smoking, and female gender (Table 3). Patients with higher stages had an HR of 1.73 (1.18–2.52), patients with high CCI scores had an HR of 1.14 (1.07–1.21), smokers had an HR of 2.07 (1.46–2.95) and male patients had an HR of 1.41 (1.01–1.96). However, age and histology were not significantly associated with prognosis (*P* > 0.05).

**Table 3:** Association between baseline characteristics and overall survival

			Univariate analysis		Multivariate analysis	
		Survival, mo (95% CI)^a^	HR (95% CI)	*P*	HR (95% CI)	*P*
Age, y	<65	20.01 (18.70–21.32)	1		1	
	≥65	18.90 (17.60–21.20)	1.24 (0.89–1.74)	0.206	0.98 (0.69–1.40)	0.922
Gender	Female	20.61 (19.35–21.86)	1		1	
	Male	18.12 (16.81–19.43)	1.41 (1.01–1.96)	0.045	1.55 (1.07–2.24)	0.021
Histology	Adenocarcinoma	19.88 (18.57–21.19)	1		1	
	Squamous	18.97 (17.69–20.25)	1.29 (0.92–1.82)	0.138	1.29 (0.88–1.89)	0.185
Cancer stage	IIIB	21.81 (19.89–23.74)	1		1	
	IV	18.60 (17.59–19.61)	1.73 (1.18–2.52)	0.005	1.36 (0.92–2.02)	0.127
CCI score	<9	20.84 (19.52–22.17)	1		1	
	≥9	17.80 (16.64–18.96)	1.14 (1.07–1.21)	<0.001	1.10 (1.02–1.17)	0.009
Smoking status	Nonsmoker	21.41 (19.82–22.99)	1		1	
	Smoker	18.10 (17.07–19.12)	2.07 (1.46–2.95)	<0.001	1.52 (1.02–2.26)	0.042

^a^Median

The multivariate analysis showed that higher CCI scores (CCI ≥ 9), smoking, and male gender were associated with higher HRs (*P* < 0.05; Table 3). Performance status was included in the multivariate analysis to assess whether there was an association with survival that was not captured by the CCI score. It was found that the HR for CCI score decreased from 1.10 to 1.07 when performance status was included in the analysis, but the association of survival with the CCI score remained significant (*P* < 0.05).

## Discussion

This study found a comprehensive prognostic factor for predicting whether NSCLC patients can benefit from chemotherapy. To rule out possible interference from other factors, we chose only patients with stage IIIB-IV (advanced, non-resectable) NSCLC without gene mutations in EGFR or ALK. The results showed that higher CCI scores (CCI ≥ 9), smoking, and male gender were associated with higher HRs.

Currently, the prognostic determinants of NSCLC are disease stage, performance status, male gender, age greater than 60 years, non-squamous histology, and weight loss. Grosso et al. demonstrated that intraoperative complications did not differ between young and old patients who underwent surgery for stage I-III colorectal cancer, whereas some differences were found in postoperative complications and late complications [26]. In another study, Grosso et al. found a significant association between a greater adherence to the Mediterranean diet (MD) and lower odds of having cancer (odds ratio = 0.46, 95% confidence interval: 0.28–0.75) [27]. Marventano et al. found that symptoms, surgical procedures and the number of comorbidities significantly affected health-related quality of life (QoL) [28]. However, recent studies suggest that these prognostic factors should be reassessed. Multicentre studies with large sample sizes have indicated that compared with age, the CCI score is a better predictor of survival for patients with advanced NSCLC [22]. Brock et al. demonstrated that comorbidities, tumour stage, and gender have a greatest influencer upon survival than age in patients with resectable NSCLC [29]. However, Janssen-Heijnen et al. concluded that comorbidity had no independent prognostic effect in elderly patients with NSCLC [30]. A study conducted by Gironés et al. showed that, although there was a high prevalence of comorbidity in untreated lung cancer patients older than 70 years, comorbidity was not related to survival [31].

In the present study, we evaluated whether the CCI score can be used to predict chemotherapy benefit and survival in patients with unresectable stage IIIB-IV NSCLC. As cancer metastasis is very common in these patients, the CCI score is usually >6. We adjusted the CCI groups using ROC analysis to determine the best cut-off (CCI = 9). We found that the CCI score, gender, and smoking status were prognostic factors for patients with unresectable stage IIIB-IV NSCLC without EGFR or ALK gene mutations. In the clinical setting, we usually choose cancer stage and performance status to determine the treatment strategy. Although these factors are easy to determine, their prognostic accuracy is questionable. Rather, the present study found that the CCI score, smoking status, and gender are potential prognostic factors for patients with unresectable stage IIIB-IV NSCLC. Although there is evidence that comorbidities may influence the progression of aggressive cancers, patients often die of the cancer before the comorbidity can affect survival [32, 33]. Our present study showed that comorbidity can indeed significantly affect survival in patients with unresectable stage IIIB-IV NSCLC without EGFR and ALK genetic mutations.

TNM staging is an important factor in the prognosis of NSCLC patients. However, for advanced NSCLC patients, such as patients with stage IIIB or stage IV NSCLC, whose tumours have similar biological behaviours, the main treatment and prognosis (unresectable tumours, mainly treatment with chemotherapy and radiotherapy) are very similar. In our study, tumour staging was significant in the univariate analysis of OS but not in the multivariate analysis, indicating that tumour staging is not an independent predictor of OS. For example, most of the chemotherapy drugs are hepatotoxic and nephrotoxic; the most commonly used chemotherapy drug, paclitaxel, isneurotoxic, and the new targeted drug bevacizumab has the side effect of arterial embolization. When IIIB patients cannot be effectively treated because of liver and kidney dysfunction, diabetes, peripheral neuropathy, cardiovascular and cerebrovascular events, and other comorbidities, the OS of these patients may lower than that of stage IV patients without these comorbidities or with less comorbidity. We found that the CCI score was more significantly associated with the prognosis of NSCLC patients than was tumour staging. This finding also shows that the presence of complications may have a certain impact on the treatment of the NSCLC patients, which may affect the prognosis of patients. Comorbidities in patients with aggressive cancers should not be ignored. Further research is needed to determine whether the CCI score is a strong prognostic factor in patients with other cancers and whether it is useful in choosing chemotherapeutics.

This study is limited, first, by the relatively small sample size, as more cases would increase the statistical power of the results. Second, the CCI was not designed specifically for patients with cancer, and some common comorbidities (such as hypertension) have not been incorporated into the CCI. A CCI score that is applicable specifically for cancer patients should be established. Finally, we defined the cut-off CCI score as9 to predict whether patients can benefit from chemotherapy, and this should be confirmed in the future.

## Conclusions

The CCI score can be considered an independent prognostic factor for stage IIIB-IV NSCLC patients that do not have EGFR or ALK genetic mutations. Performance status attenuates the strength of the association with the CCI score, but the CCI score is still significantly associated with survival after adjusting for performance status. Smoking status and gender were also found to be independent prognostic factors in this study. More large-scale research is required to further verify whether the CCI score is an independent prognostic factor for the overall survival of patients with NSCLC and other solid tumours.

## Abbreviations

ALK: Anaplastic lymphoma kinase
CCI: Charlson comorbidity index
EGFR: Epidermal growth factor receptor
NSCLC: Non-small cell lung cancer
